# Supplementary material for: Metabolic Profile of C-Prenyl Coumarins Using Mass Spectrometry-Based Metabolomics
Source: Molecules. 2021 Oct 29;26(21):6558. doi: 10.3390/molecules26216558 (PMC8588418; doi:10.3390/molecules26216558)
Supplement: Supplementary file 1 [file molecules-26-06558-s001.zip › molecules-1431107-supplementary.pdf]

Supplementary Materials

Metabolic Profile of C-Prenyl Coumarins Using Mass Spectrometry-Based Metabolomics

Yan Cheng[1,#], Xiao-Fang Ma[2,#], Qi Zhao[1], Chun-Yan Wang[1]
Dong-Mei Yan[2,], Fei Li[1]

1 Laboratory of Metabolomics and Drug-induced Liver Injury, Frontiers Science Center for Disease-related Molecular Network, West China Hospital, Sichuan University, Chengdu 610041, China; Yancheng2020@wchscu.cn

2 Academician Workstation, Jiangxi University of Chinese Medicine, Nanchang 330004, China; yanli1999@163.com

[#] These authors equally contributed to this work.

Figure S1. Metabolic profiling of MH, ISM and MER *in vitro*. (A) Scores plot of OPLS-DA and S-plot analysis from control and MH incubated with MLM. **(B)** Scores plot of OPLS-DA and S-plot analysis from control and ISM incubated with MLM. **(C)** Scores plot of OPLS-DA and S-plot analysis from control and MER incubated with MLM.

Figure S2. Role of MH, ISM and MER in RAW 264.7. (A) Cell viability test of MH, ISM and MER in RAW 264.7. **(B)** Correlation of NO production and inflammatory cytokines transcription level.

Figure S3. Metabolic map of MH and ISM. (A) Metabolic map of MH. **(B)** Metabolic map of ISM.

Fig S1

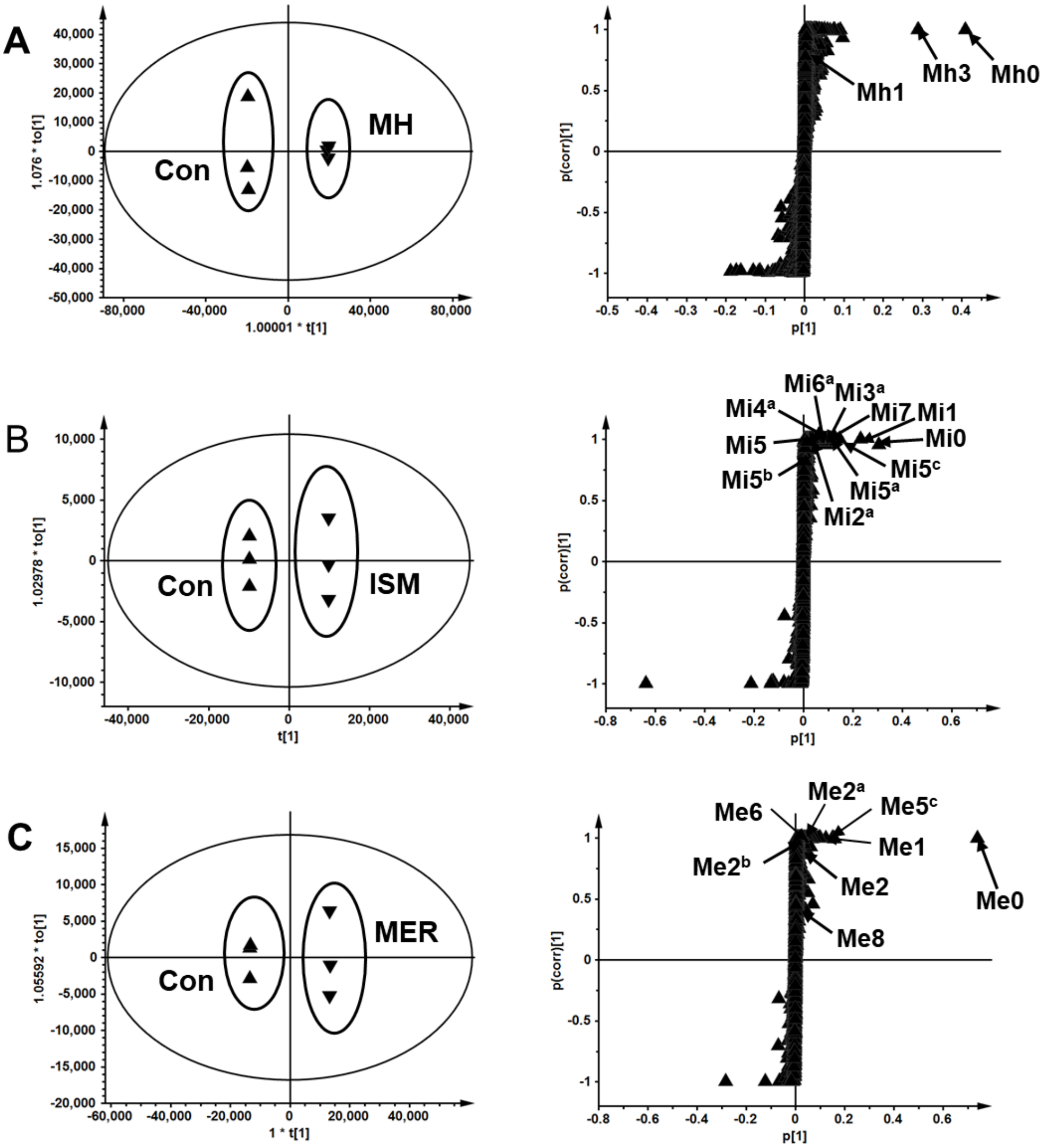

A
B
C
Con
MH
ISM
MER
Mh0
Mh1
Mh3
Mi0
Mi1
Mi2a
Mi3a
Mi4a
Mi5
Mi5a
Mi5b
Mi5c
Mi6a
Mi7
Me0
Me1
Me2
Me2a
Me2b
Me5c
Me6
Me8
1.00001 * t[1]
1.076 * to[1]
t[1]
1.02978 * to[1]
1 * t[1]
1.05592 * to[1]
p[1]
p(corr)[1]

Fig S2

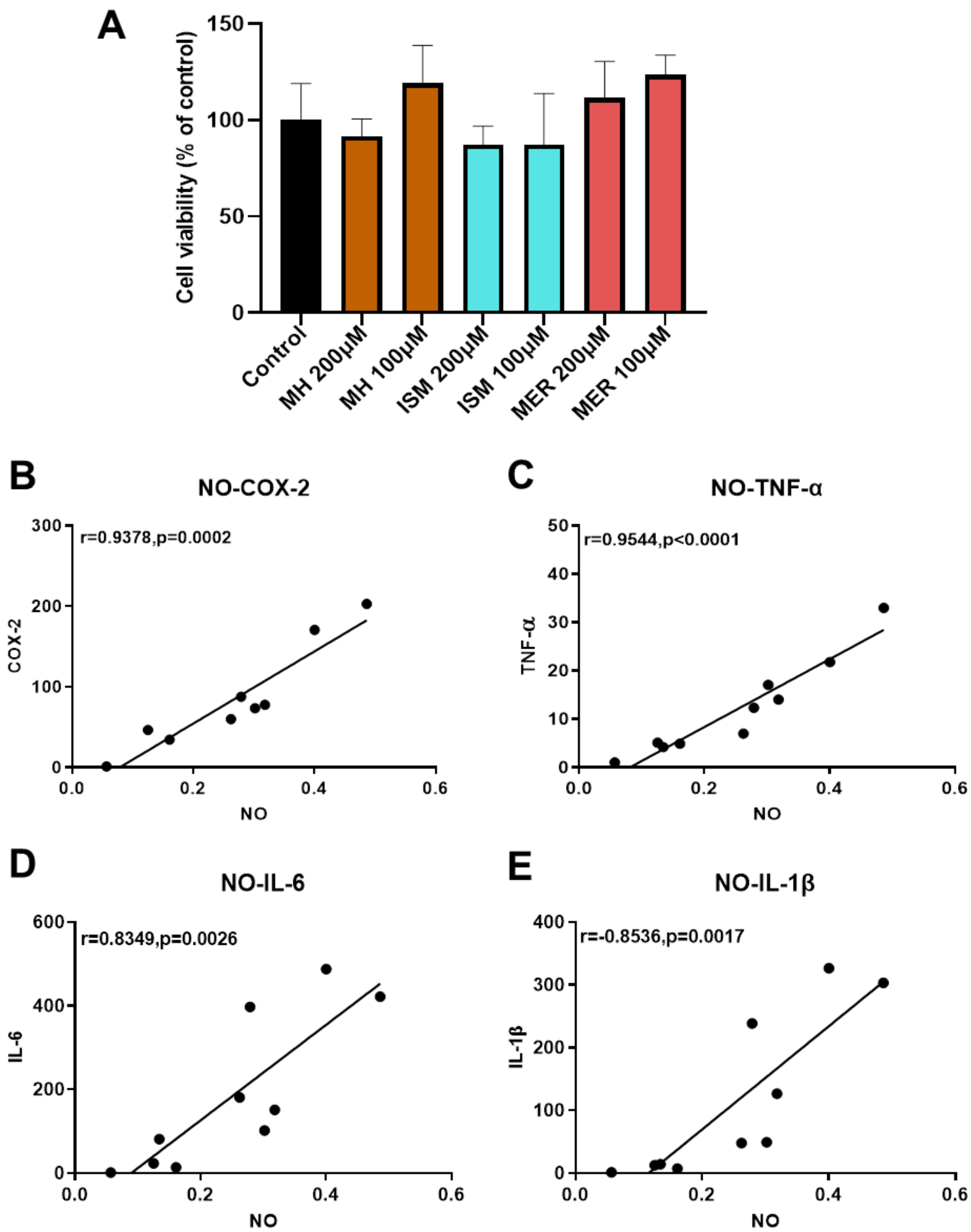
A
Cell viability (% of control)
150
100
50
0
Control
MH 200μM
MH 100μM
ISM 200μM
ISM 100μM
MER 200μM
MER 100μM
B
NO-COX-2
r=0.9378,p=0.0002
COX-2
NO
C
NO-TNF-α
r=0.9544,p<0.0001
TNF-α
NO
D
NO-IL-6
r=0.8349,p=0.0026
IL-6
NO
E
NO-IL-1β
r=-0.8536,p=0.0017
IL-1β
NO

Fig S3

A

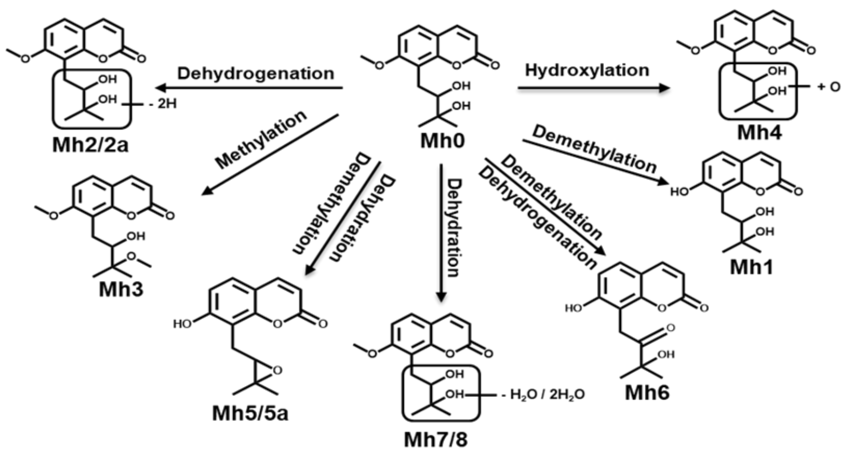
Dehydrogenation
Hydroxylation
Methylation
Demethylation
Dehydration
Demethylation Dehydration
Demethylation Dehydrogenation
Mh0
Mh1
Mh2/2a
Mh3
Mh4
Mh5/5a
Mh6
Mh7/8
- 2H
+ O
- H2O / 2H2O

B

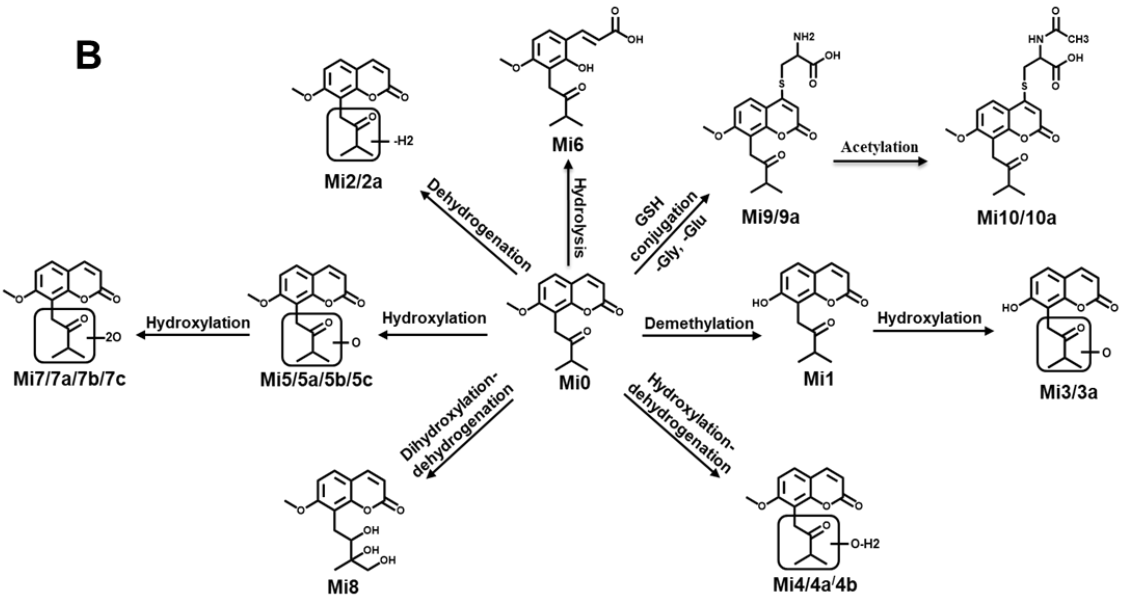
Hydrolysis
Dehydrogenation
GSH conjugation -Gly, -Glu
Acetylation
Hydroxylation
Demethylation
Dihydroxylation-dehydrogenation
Hydroxylation-dehydrogenation
Mi0
Mi1
Mi2/2a
Mi3/3a
Mi4/4a/4b
Mi5/5a/5b/5c
Mi6
Mi7/7a/7b/7c
Mi8
Mi9/9a
Mi10/10a

Table S1. Summary of MH metabolites produced *in vivo* and *in vitro* metabolism

**Metabolite ID**	**Molecular formula**	**RT (min)**	**m/z[M+H]$^+$**	**Mass error (ppm)**	**MS/MS fragments**	**Identification**	**Source**
Mh0	$C_{15}H_{18}O_5$	6.32	279.1227	2.53	261, 243, 189, 175, 159	Meranzin hydrate	C, H, M
Mh1*	$C_{14}H_{16}O_5$	5.57	265.1070	1.64	247, 229, 223, 177	Mh-CH_2	C, M
Mh2*	$C_{15}H_{16}O_5$	5.40	277.1070	3.81	259, 249, 235, 227, 219	Mh-2H	C, M, U
Mh2a*	$C_{15}H_{16}O_5$	6.07	277.1070	-1.24	259, 231, 205, 189, 175	Mh-2H	H, M
Mh3*	$C_{16}H_{20}O_5$	6.12	293.1383	-4.92	275, 261, 247, 229, 217	Mh+CH_2	H, M, U
Mh4*	$C_{15}H_{18}O_6$	6.05	295.1176	-1.08	277, 259, 247, 219, 205	Mh+O	M
Mh5*	$C_{14}H_{14}O_4$	3.98	247.0960	3.64	265, 229, 201, 187, 175	Mh-CH_2-H_2O	U
Mh5a*	$C_{14}H_{14}O_4$	5.05	247.0960	3.64	263, 229, 201, 187, 175	Mh-CH_2-H_2O	U
Mh6*	$C_{14}H_{14}O_5$	4.51	263.0910	-2.66	245, 277,191, 163, 116	Mh-CH_2-2H	U
Mh7*	$C_{15}H_{14}O_3$	5.84	243.1010	4.93	228, 203, 189, 175,159	Mh-2H_2O	U
Mh8*	$C_{15}H_{16}O_4$	5.81	261.1110	-4.59	243, 189, 177, 159, 131	Mh-H_2O	P

a/b/c/d, isomer metabolite; *, undescribed metabolite. P, plasma; U, urine; C, cell; H, HLM; M, MLM;

Table S2. Summary of ISM metabolites produced *in vivo* and *in vitro* metabolism

**Metabolite ID**	**Molecular formula**	**RT (min)**	**m/z[M+H]$^+$**	**Mass error (ppm)**	**MS/MS fragments**	**Identification**	**Source**
Mi0	$C_{15}H_{16}O_4$	8.41	261.1121	1.80	243, 231, 201, 189	Isomeranzin	P, F, U, H, M
Mi1*	$C_{14}H_{14}O_4$	6.94	247.0965	3.32	229, 201, 187, 175	Ism-CH_2	P, F, U, H, M
Mi2*	$C_{15}H_{14}O_4$	7.11	259.0965	-6.10	243, 231, 213, 189	Ism-2H	H, M
Mi2a*	$C_{15}H_{14}O_4$	8.19	259.0965	-1.85	241, 231, 205, 189	Ism-2H	F, U, H, M
Mi3*	$C_{14}H_{14}O_5$	5.54	263.0914	1.54	245, 233, 221, 187	Ism+O-CH_2	F, M
Mi3a*	$C_{14}H_{14}O_5$	6.39	263.0914	3.06	245, 217, 203, 191, 175	Ism+O -CH_2	H, M
Mi4*	$C_{15}H_{14}O_5$	6.08	275.0914	-8.70	231, 215, 203, 189	Ism+O -2H	H
Mi4a*	$C_{15}H_{14}O_5$	6.79	275.0914	1.47	259, 247, 231, 217, 205	Ism+O -2H	H
Mi4b*	$C_{15}H_{14}O_5$	7.58	275.0914	2.56	259, 247, 243, 217	Ism+O-2H	M
Mi5*	$C_{15}H_{16}O_5$	6.33	277.107	0.56	263, 247, 231, 221	Ism+O	U, H, M
Mi5a*	$C_{15}H_{16}O_5$	6.67	277.107	3.45	259, 231, 217, 205, 189	Ism+O	H, M
Mi5b*	$C_{15}H_{16}O_5$	7.12	277.107	-0.88	259, 249, 243, 233, 227	Ism+O	F, H, M
Mi5c*	$C_{15}H_{16}O_5$	7.95	277.107	3.56	259, 243, 221, 205,	Ism+O	F, U, H, M
Mi6*	$C_{15}H_{18}O_5$	6.99	279.1227	5.03	261, 233, 249, 219	Ism+H_2O	U
Mi7*	$C_{15}H_{16}O_6$	5.71	293.102	2.18	275, 263, 245, 231, 217	Ism+2O	H, M
Mi7a*	$C_{15}H_{16}O_6$	6.08	293.102	10.37	279, 263, 219, 203, 189	Ism+2O	H
Mi7b*	$C_{15}H_{16}O_6$	6.36	293.102	15.83	275, 261, 247, 233, 207	Ism+2O	M
Mi7c*	$C_{15}H_{16}O_6$	6.63	293.102	4.23	277, 259, 231, 217, 205	Ism+2O	M
Mi8*	$C_{15}H_{18}O_6$	6.24	295.1176	0.99	277, 259, 233, 221, 203	Ism+2O+2H	H, M
Mi9*	$C_{18}H_{21}NO_6S$	5.36	380.1162	-0.08	334, 291, 259	Ism+Cysteine	P, F, U
Mi9a*	$C_{18}H_{21}NO_6S$	6.04	380.1162	2.56	362, 334, 291, 259	Ism+Cysteine	P, F, U
Mi10*	C20H23NO7S	6.56	422.1268	0.72	376, 334, 291, 259	Ism+*N*-acetylCysteine	F, U
Mi10a*	$C_{20}H_{23}NO_7S$	7.36	422.1268	2.15	404, 380, 376, 362, 334	Ism+*N*-acetylCysteine	F, U

a/b/c/d, isomer metabolite; *, undescribed metabolite. P, plasma; F, feces; U, urine; H, HLM; M, MLM
